# Active packaging coating based on *Lepidium sativum* seed mucilage and propolis extract: Preparation, characterization, application and modeling the preservation of buffalo meat

**DOI:** 10.1371/journal.pone.0311802

**Published:** 2024-10-09

**Authors:** Fatemehe Majdi, Behrooz Alizadeh Behbahani, Hassan Barzegar, Mohammad Amin Mehrnia, Morteza Taki

**Affiliations:** 1 Faculty of Animal Science and Food Technology, Department of Food Science and Technology, Agricultural Sciences and Natural Resources University of Khuzestan, Mollasani, Iran; 2 Faculty of Agricultural Engineering and Rural Development, Department of Agricultural Machinery and Mechanization Engineering, Agricultural Sciences and Natural Resources University of Khuzestan, Mollasani, Iran; Benha University, EGYPT

## Abstract

Buffalo meat is naturally perishable, making it susceptible to spoilage due to its high moisture content and vulnerability to microbial contamination. Edible coatings have attracted attention as a packaging method that can prolong the shelf life of meat. The study aimed to examine the impact of a combination of *Lepidium sativum* mucilage (LS) coating and propolis extract (PE) on prolonging the shelf life of buffalo meat. The chemical characteristics (chemical compounds, total phenol content (TPC), total flavonoid content (TFC), antioxidant activity, and cytotoxicity) and antimicrobial activity of the PE (disk diffusion agar, well diffusion agar, minimum inhibitory concentration, and minimum bactericidal concentration) were investigated. The effect of the PE on the cell wall of pathogenic bacteria was examined using a scanning electron microscope. Biological properties of LS (TPC, TFC, antioxidant activity and antimicrobial effect (pour plate method)) was investigated. Different concentrations of PE (0, 0.5, 1.5, and 2.5%) were added to the coating mixture containing LS, and their effects on extending the shelf life of buffalo meat samples stored at 4°C for 9 days were assessed. The PE included gallic acid, benzoic acid, syringic acid, 4–3 dimethoxy cinnamic acid, *p*-coumaric acid, myricetin, caffeic acid, luteolin, chlorogenic acid, and apigenin. The PE was determined to have a TPC of 36.67 ± 0.57 mg GAE/g and a TFC of 48.02 ± 0.65 mg QE/g. The extract’s radical scavenging activity ranged from 0 to 76.22% for DPPH radicals and from 0 to 50.31% for ABTS radicals. The viability of C115 HeLa cell was observed to be 94.14 μg/mL. The PE and LS, exhibited strong antimicrobial properties against pathogenic bacteria. The LS was determined to have a TPC of 15.23 ± 0.43 mg GAE/g and a TFC of 11.51± 0.61 mg QE/g. The LS was determined to have a DPPH of 429.65 ± 1.28 μg/mL and a ABTS of 403.59 ± 1.46 μg/mL. The microbiological analysis revealed that the LS+2.5%PE treatment was the most effective in inhibiting the growth of total viable count (6.23 vs. 8.00 log CFU/g), psychrotrophic bacteria count (3.71 vs. 4.73 log CFU/g), coliforms count (2.78 vs. 3.70 log CFU/g), and fungi count (2.39 vs. 3.93 log CFU/g) compared to the control sample. The addition of PE to the edible coating also demonstrated a concentration-dependent effect on preserving the moisture, pH, color, and hardness of the buffalo meat. Sensory evaluation results suggested that incorporating PE into the edible coating extended the shelf life of buffalo meat by three days. In the second stage of this paper, this investigation employed two distinct forecasting methodologies: the Radial Basis Function (RBF) and the Support Vector Machine (SVM), to predict a range of quality indicators for coated meat products. Upon comparison, the RBF model exhibited a higher level of accuracy, showcasing its exceptional capacity to closely match the experimental outcomes. Therefore, this type of food coating, renowned for its strong antimicrobial properties, has the potential to effectively package and preserve perishable and delicate food items, such as meat.

## 1. Introduction

Buffalo meat is esteemed as a valuable protein source in numerous global cultures, revered for its savory taste and nutritional advantages [1,2]. Nonetheless, buffalo meat is naturally perishable, making it susceptible to spoilage due to its high moisture content and vulnerability to microbial contamination [3,4]. The perishable quality of buffalo meat presents a substantial hurdle in upholding its quality and safety during storage and transportation [5]. Implementing effective preservation techniques is crucial to prolong the shelf life of buffalo meat and guarantee its availability for consumption [2,3,6].

Packaging of food is essential for maintaining the freshness and quality of perishable food products [7,8]. Edible coatings have attracted attention as a packaging method that can prolong the shelf life of food items [7]. These coatings form a protective layer around the food, reducing moisture loss, preventing microbial growth, and slowing down oxidation processes [9–12]. By integrating active ingredients like antimicrobial agents or antioxidants, edible coatings can improve the preservation of food products and help reduce food waste [13–15].

The mucilage from *Lepidium sativum* seeds, obtained from garden cress, is a natural polysaccharide with the ability to form films, making it appropriate for edible coating purposes. This biodegradable and edible substance presents an eco-friendly substitute for synthetic coatings, offering a protective film that aids in preserving the quality and freshness of food items [16,17]. *L*. *sativum* mucilage (LS) has demonstrated promise in extending the shelf life of perishable foods by creating a barrier against moisture loss and microbial contamination [18–21].

Propolis extract (PE), a resinous substance gathered by bees from plants, is recognized for its antimicrobial and antioxidant characteristics [22–24]. The integration of PE into edible coatings has displayed potential in prolonging the shelf life of food items by suppressing microbial growth and oxidative reactions [25–28]. PE provides a natural and efficient method for preserving perishable foods while upholding their quality and safety.

The development of Artificial Neural Networks (ANN) within the realm of food engineering marks a pivotal advancement, catalyzing both innovation and operational efficiency [29]. The prowess of ANNs in deciphering intricate non-linear interdependencies renders them indispensable across a spectrum of food engineering applications, from scrutinizing quality to refining process efficacy [30]. For instance, ANNs have been harnessed to bolster the quality and safety of diverse food products, encompassing meats and dairy assortments [31] such as yogurt and cheese [32]. The study highlighted the proficiency of ANNs in reducing laboratory costs while ensuring accurate and dependable prognostications. In a distinct study, ANNs were employed to predict a variety of quality parameters for pistachios subjected to probiotic treatments, as reported by Zibaei-Rad et al. (2024) [33]. The scope of these parameters extended from visual and olfactory attributes to overall consumer receptivity, encompassing nutritional elements like carotenoids and chlorophylls, microbial profiles, antioxidative capacity, phenolic concentration, viability, soluble carbohydrates, peroxide value (PV), fat content, total soluble solids, and alterations in mass. The findings revealed a significant alignment between empirical observations and ANN projections, corroborating the model’s precision in forecasting.

The primary objective of this study is to examine the impact of using PE-loaded LS-based edible coating on the preservation of buffalo meat. By harnessing the antimicrobial properties of PE and the film-forming attributes of LS, this research aims to create a sustainable and efficient preservation technique for buffalo meat. The study is concentrated on assessing the effects of the edible coating on microbial growth, physical stability, and overall quality of buffalo meat during storage, contributing to the advancement of innovative approaches for prolonging the shelf life of perishable food products. In the second stage of the paper, two well-known models used for predicting some of properties of the coated buffalo meat to decrease the cost of laboratory analysis in the future similar studies.

## 2. Materials and methods

### 2.1. Materials

The chemicals utilized in this research were of analytical quality and were procured from Merck Co. (Germany) or Sigma-Aldrich Co. (USA). The research commenced on February 2023. Data analysis and report finalization were completed by May, 2024. Data collection was conducted in a laboratory of food microbiology, Department of Food Science and Technology in Agricultural Sciences and Natural Resources University of Khuzestan, Iran. The research did not involve minors or clinical trials.

### 2.2. Phenolic compounds

The composition of LE was identified and quantified using high-performance liquid chromatography (HPLC), following the procedure outlined by Terpinc et al. (2016) [34]. The HPLC system consisted of a binary pump (Agilent 1100), an autosampler, and a mass spectrometer with an electrospray ionizer source (MS; ESI-; Micromass Quattro Micro; Waters, Milford, MA, USA). A reversed-phase Kinetex C18 column (100 × 2.00 mm; 2.6 μm) was used, and the parameters included a capillary voltage of 3.0 kV, a cone voltage of 20 V, and an extractor of 2 V. The source temperature was maintained at 100°C, and the desolvation temperature was set at 350°C. The cone gas flow and desolvation gas flow were 30 L/h and 350 L/h, respectively. The mobile phase was a combination of 0.1% formic acid (A) and acetonitrile (B), mixed in varying proportions based on specific time intervals. The flow rate was 0.30 mL/min, and the injection volume was 10 μL. The gradient elution profile was as follows: 10% B from 0–2 min, 10–60% B from 2–20 min, 60–80% B from 20–21 min, 80% B from 21–25 min, 80–10% B from 25–26 min, and 10% B from 26–30 min.

### 2.3. Total phenolic content (TPC)

In brief, 20 μL of the PE (1 mg/mL) were successively mixed with 100 μL of Folin Ciocalteau’s phenol reagent and 300 μL of distilled water. After a 4-minute interval, 400 μL of 20% sodium carbonate and 1000 μL of distilled water were added. Subsequently, the reaction mixture was left in the dark at 25°C for 2 h, and its absorbance was read at 760 nm. The TPC was calculated using a calibration curve generated from standard solutions of gallic acid (0.5–20 μg/mL). The results were reported as milligrams of gallic acid equivalent (mg GAE) per gram of extract (mg GAE/g dry extract) [35].

### 2.4. Total flavonoids content (TFC)

The TFC was assessed utilizing the aluminum colorimetric technique, employing quercetin as the reference standard. To begin, a 100 μL portion of PE (1 mg/mL) was systematically combined with 2% (w/v) AlCl_3_ (200 μL), 3.5 mL of water, 1 mL of methanol, and 1 M potassium acetate (200 μL). Subsequently, the mixture was left to incubate at room temperature for a 30-minute interval prior to measuring the absorbance at 435 nm. The TFC was subsequently represented as milligrams of quercetin equivalent per gram of dry weight of the extract (mg QE/g) [35].

### 2.5. Fourier transform infrared (FTIR) spectroscopy

The functional groups of PE were analyzed using FTIR spectroscopy. After being mixed with potassium bromide, the extract was compressed into a tablet form. Subsequently, the sample was scanned using an FTIR spectrophotometer (Perkin Elmer, USA) within the range of 400–4000 cm^−1^ [36].

### 2.6. Antioxidant activity

In this research, the antioxidant activity of the PE was evaluated using DPPH (2 2-diphenyl-1-picrylhydrazyl) and ABTS (2,2’-azino-bis(3-ethylbenzothiazoline-6-sulfonic acid)) radical scavenging (RS) assays [37]. To prepare the DPPH^•^ solution, 0.3 mM DPPH^•^ was dissolved in 96% ethanol. PE, or ethanol (as a control) was mixed with the DPPH^•^ solution, and the decrease in absorption at 517 nm was recorded. For the ABTS assay, a 7 mM ABTS solution was prepared in water. ABTS radical cation (ABTS^•**+**^) was generated by reacting ABTS stock solution with 2.45 mM potassium persulfate and allowing the mixture to stand at room temperature for 16 h. After mixing standard solution or PE with the ABTS radical cation solution, the absorbance of the sample was measured at 730 nm to determine the antioxidant activity.


$$Radical\;scavenging\;activity=(1‐A_{sample}/A_{control})\times 100$$

### 2.7. Antibacterial effect

The study conducted by Alizadeh Behbahani et al. (2018) [38] assessed the antimicrobial properties of PE against *Escherichia coli*, *Pseudomonas aeruginosa*, *Bacillus cereus*, *Listeria innocua*, *Staphylococcus aureus*, and *Salmonella typhi*. This evaluation was carried out using disc diffusion agar (DDA), well diffusion agar (WDA), minimum inhibitory concentration (MIC), and minimum bactericidal concentration (MBC) methods, with some modifications.

In the DDA experiment, PE was prepared in concentrations of 32, 64, 128, 256, and 512 mg/mL (distilled water), and then sterilized using a 0.22 μm syringe microfilter. The prepared disks were soaked in these solutions for 15 min. A sterile swab was used to spread the microbial suspension on the culture plates, and then the prepared disks were placed on the surface of Mueller Hinton agar (MHA) medium. Following incubation at 37°C for a duration of 24 h, the plates were then subjected to assessment to determine the antimicrobial efficacy by gauging the size of the inhibition zone (IZ) in millimeters. To serve as a reference point in this study, chloramphenicol antibiotic was utilized as the positive control.

In the WDA method, MHA medium was poured into a plate and an L-shaped spreader was utilized to evenly spread the microbial suspension on the MHA medium. Subsequently, 6 mm-wide wells were meticulously crafted on the surface of the culture medium. Diverse concentrations of the extract, spanning from 32, 64, 128, 256, and 512 mg/mL, were carefully introduced into these wells, following which the cultures were incubated for a period of 24 h at 37°C. Finally, IZs around wells were measured in millimeters and recorded.

To determine the MIC, the following procedure was carried out: initially, a culture containing 1.5 × 10^8^ colony forming unit (CFU)/mL of bacteria was prepared. Subsequently, an extract solution was prepared in dimethyl sulfoxide (DMSO) solution, which was then diluted with Mueller Hinton broth (MHB). 125 μL of the microbial suspension was added to each well of the 96-well plate, and the plate was then incubated at 37°C for 24 h. Following incubation, 25 μL of triphenyltetrazolium chloride reagent solution (5 mg/mL) was added to each well. A deep red or amethystine color developed in wells containing microbial growth in less than 30 min. The MIC was identified as the lowest concentration at which no microbial growth or color change was observed. To determine the MBC, 100 μL of the culture medium from wells without red color on the plate was plated on MHA and incubated at 37°C for 24 h. The MBC was defined as the minimum dilution that inhibited colony formation.

### 2.8. Scanning electron microscopy (SEM)

The bacterial strains’ structural changes were assessed using SEM, according to a method described by Jalil Sarghaleh et al. (2023) [36]. The experiment began with centrifuging the microbial suspension at 5000 × g for 5 min to separate the *E*. *coli* and *S*. *typhi* strains. The microbial suspension was then washed with 100 mM sodium phosphate buffer at pH 7 and filtered. The dissolved microbial sample was stabilized with 2.5% v/v glutaraldehyde and incubated at 4°C for 2 h. Final dehydration and washing steps were conducted using distilled water and ethanol, respectively. Subsequently, the sample was dried in a vacuum, coated with a layer of gold, and examined using an SEM apparatus (VP model, Germany).

### 2.9. Cytotoxic activity

The study involved assessing the toxicity of PE on the C115 HeLa cell line using the MTT (3-(4,5-dimethylthiazol-2yl)-2,5 diphenyl tetrazolium bromide) protocol, which is a method for measuring cell proliferation. The cells were cultured in Dulbecco’s Modified Eagle Medium (DMEM) supplemented with fetal bovine serum and penicillin/streptomycin. Subsequently, they were placed in a carbon dioxide incubator at 37°C with 95% humidity. 100,000 cells were seeded in each well of a 96-well plate, and a solution containing varying concentrations of PE (0, 10, 25, 50, 100, and 200 mg/mL) along with 200 μL of fetal bovine serum was added to the DMEM culture medium. After 24 h of incubation, MTT solution was added to each well and incubated for 3 h in a carbon dioxide incubator. Following this, the medium was aspirated, and DMSO was added to each well. Finally, the absorbance at a wavelength of 570 nm was measured using an ELISA reader (ELX 808, Bio Tek Instruments, USA), and cell survival curves were plotted using control cells [36].

### 2.10. Biological properties of *Lepidium sativum* mucilage (LS)

#### 2.10.1. Antimicrobial effect of *Lepidium sativum* mucilage (LS)

The pour plate method (PPM) is a qualitative technique utilized for assessing the antimicrobial efficacy of LS. In the experiment described, 0.2 g of LS were transferred to a sterile 10 mL test tube and mixed with 5 mL of distilled water. Subsequently, a 1 mL portion of this solution was evenly distributed onto petri dishes containing MHA medium (19 mL). The agar plates were then incubated at the appropriate temperature for bacterial growth (at 37°C for a duration of 24 h), allowing the LS to exert its antimicrobial effects. After the specified incubation period, the plates were examined for the presence and extent of bacterial growth. The results of this method categorize the impact as sensitive, semi-sensitive, or resistant depending on the observed bacterial growth on agar plates [38,39].

#### 2.10.2. Determination of Total flavonoids content (TFC) and Total phenolic content (TPC) of *Lepidium sativum* mucilage (LS)

The method involving aluminum chloride was utilized to determine the TFC of LS. Various concentrations of LS were mixed with methanolic aluminum chloride 2%, followed by incubation at room temperature in darkness for 15 minutes. The absorbance value was then measured at 430 nm using a spectrophotometer (Sigma3-30k). This colorimetric assay was also employed to determine the TPC [40].

#### 2.10.3. Determination of antioxidant activity of *Lepidium sativum* mucilage (LS)

Initially, 1 mL of a LS solution at a concentration of 1 mg/mL was combined with 4 mL of 0.1 mM DPPH methanolic solution. The tubes were then gently shaken and left in the dark for 20 minutes at room temperature. Subsequently, the optical density (OD) of the mixture was measured at 517 nm using a UV spectrometer (UV-1800, Shimadzu). The calculation of free radical scavenging activity was performed using the specified equation [41].


$$Radical\;scavenging\;activity\;(\%)=\frac{control\;OD-sample\;OD}{control\;OD}\times 100$$

The ABTS+ radical scavenging activity of mucilage was determined following the method outlined by Oh et al. (2022) [41] with certain modifications. To generate ABTS^•+^, equal volumes of 7 mM ABTS stock solution and 2.45 mM potassium persulfate solution were mixed and allowed to stand in darkness at 0°C for 12–16 hours. The resulting ABTS^•+^ solution was then diluted with ethanol until the absorbance reached 0.700 ± 0.20 at 734 nm. For the experimental procedure, 0.2 mL of the LS solution (1 mg/mL) was mixed with 1.8 mL of the ABTS^•+^ solution. After a 10-minute incubation period in darkness, the absorbance was measured at 734 nm. A control mixture of 0.2 mL ethanol with 1.8 mL of ABTS^•+^ solution was used for comparison. The results of antioxidant activity were reported based on IC_50_. The IC_50_ value is defined as the concentration of test compounds that can inhibit free radicals by as much as 50% [41].

### 2.11. Preparation of edible coating and buffalo meat coating

Obtaining LS from the plant seeds involved an extraction process with a water to seed ratio of 30:1, a pH level of 10, and a temperature set at 35°C for a duration of 15 min. Post the meticulous removal of mucilage from the seed exteriors, the material underwent filtration and was subsequently dried overnight at 60°C. The resultant mucilage was then meticulously processed, packaged, and stored at ambient temperature, following the procedures outlined by Karazhiyan et al. (2011) [42].

For the formulation of the edible coating, 2 g of LS and 0.1 g of Tween 80 were dissolved in 100 mL of sterile distilled water, with subsequent stirring and heating for 2 h. Various concentrations of PE (0, 0.5, 1.5, and 2.5% v/v) were then integrated into the hydrocolloidal solution (2%) to yield a biologically active edible coating endowed with both antioxidant and antimicrobial properties, as per the methodology by Barzegar et al., (2020) [18].

Buffalo meat sourced from a nearby establishment in Ahvaz, Iran, was acquired within 24 h post slaughter and subsequently sectioned into uniform pieces measuring 2 × 2 × 2 cm^3^, with an average weight of around 5 ± 0.1 g each. These portions were randomly divided into five distinct groups and subjected to different treatments for a duration of 3 min, followed by a 5-minute period of rest at room temperature to allow the processes to take effect. Post-treatment, the samples were positioned to facilitate drainage of any surplus solution, after which they were refrigerated at 4°C for a period of 9 days in accordance with the experimental protocol detailed by Rouhi et al. (2024) [43]. The specific treatments included: control sample (non-coated), LS (sample coated with LS), LS+0.5%PE (sample coated with LS containing 0.5% PE), LS+1.5%PE (sample coated with LS containing 1.5% PE), and LS+2.5%PE (sample coated with LS containing 2.5% PE).

#### 2.11.1. Microbiological analysis

To prepare the buffalo meat samples for testing, 5 g of the samples were combined with 45 mL of 0.1% peptone water. This mixture was then homogenized at 200 rpm for 1 min using a Stomacher. Dilutions were then made using the same peptone water, with subsequent dilutions prepared up to 10^−9^. The petri dishes were then filled with culture media (20 mL) and inoculated with the diluted samples. The microbial tests conducted included the total viable count, which was incubated at 37°C for 48 h using plate count agar, psychrotrophic bacteria count, which was incubated for 10 days at 7°C using plate count agar, coliforms count, which was incubated for 24 h at 37°C using violet red bile agar, and fungi count, which was incubated at 27°C for 72 h using sabouraud dextrose agar [3,43].

### 2.12. Moisture content

The oven drying method was applied to measure the moisture content of the buffalo meat samples [44].

### 2.13. pH

In order to determine the pH levels, 5 g of the meat samples were thoroughly blended with 45 mL of sterile water for a period of 30 s. Subsequently, the pH of the resultant homogenate was gauged using a digital pH meter [44].

### 2.14. Hardness

The buffalo meat samples’ hardness changes during cold storage were evaluated using a stable microsystem texture analyzer (TA, XT2i, UK). The 2 × 2 × 2 cm^3^ samples were compressed with a cylindrical probe of 36 mm diameter at a test speed of 5 mm/s, compressing them to 50% of their initial height. The maximum force (N) exerted during compression was recorded as the sample texture’s hardness [45].

### 2.15. Color

The color characteristics of the meat samples, including lightness (L*), redness (a*), and yellowness (b*), were assessed using a CR-400 colorimeter (Konica Minolta, Japan). The total color difference (ΔE), hue angle (*h*°) and chroma index (C*) or saturation index among the samples was determined using the equation below [4,46].


$$\mathrm{\Delta E}=\sqrt{(\Delta L*{)}^{2}+(\Delta b*{)}^{2}+(\Delta a*{)}^{2}}$$

$$hue\;angle\;(h{}^{\circ})=arctan\;(\frac{b*}{a*})$$

$$Chroma\;index\;(C*)=\sqrt{a^{*2}+b^{*2}}$$

### 2.16. Sensory properties

This sensorial analysis was conducted with utmost consideration for ethical principles and guidelines. It started on Jun 12, 2023, and ended on August 20, 2023. The participants in the study were fully informed about the nature and purpose of the analysis, and their voluntary consent was obtained prior to their involvement. Confidentiality and anonymity of the participants were strictly maintained throughout the analysis. The study adhered to all relevant ethical regulations and guidelines to ensure the well-being, safety, and rights of the participants. Any potential risks or discomfort to the participants were minimized, and appropriate measures were taken to ensure their welfare. The data collected during the analysis will be handled with confidentiality and used solely for research purposes. The sensory attributes of the samples were assessed utilizing the 9-point hedonic scale method. A panel comprising 25 semi-trained assessors was tasked with evaluating the samples based on characteristics such as odor, color, texture, and overall acceptance, utilizing a rating scale ranging from 1 (indicative of very unpleasant) to 9 (indicative of very favorable). Each assessor was presented with five samples or treatments of buffalo meat arranged in small porcelain containers. It’s important to note that the assessors were kept unaware of the specific experimental conditions, and the samples were coded in a blinded fashion. Samples that garnered a score exceeding 4 points were deemed acceptable [47].

### 2.17. Statistical analysis

The data collected was subjected to analysis utilizing Minitab software (version 16), employing a completely randomized design in factorial arrangement. For enhanced reliability, the experiments were replicated three times. Subsequently, the means were clustered utilizing the Tukey post-test method with a significance threshold set at p < 0.05 to ascertain any statistically significant differences among the various treatments or samples.

### 2.18. Support Vector Machine (SVM)

The foundational work on SVM methods was widespread adoption due to their compelling attributes and robust experimental results [48]. Initially conceived for classification tasks, SVMs quickly became a tool of choice for regression problems as well [49]. The mathematical formulation of Support Vector Regression (SVR) is predicated on the principle of structural risk minimization, and it commences with the dataset represented as [50]:

$$s=\{\left(x_{1},y_{1})\ldots \ldots (x_{n},y_{n}\right)\}$$

Where, (*x*_*i*_, *y*_*i*_) *x*_*i*_∈*R*^*d*^ *y*_*i*_∈*R* and any desired value is the output for the input vector. The regression model is trained in these patterns and uses the neglected input vector to predict the desired target value [51].

In order for a stronger learning method to be developed, input data does not need to be placed exactly on or inside tube *ε*. Instead, data that is out of range is fined and slack variables are replaced in such cases. The target function and constraints are usually given as [52]:

$$\begin{array}{l} \frac{1}{2}\langle w,w\rangle +c\sum_{i=1}^{n}\left(\xi_{i}+\xi_{i}^{*}\right),\\ (\langle w,\phi (x_{i})\rangle +b)-y_{i}\leq \varepsilon +\xi_{i},\\ y_{i}-(\langle w,\phi (x_{i})\rangle +b)\leq \varepsilon +\xi_{i}^{*},\\ \xi_{i},\xi_{i}^{*}\geq 0,i=1,\ldots ,n, \end{array}$$

Where, C is a parameter that balances the complexity of the model and the training errors, *ξ*_*i*_ and *ξ*_*i*_ are the inert variables for the target value, respectively [53]. (*ξ*−*ε*) is defined as:

$${\left|\xi \right|}_{\varepsilon }=\{\begin{array}{c} 0|\xi |\leq \varepsilon \\ |\xi |-\varepsilon \end{array}$$

**Fig 1:**
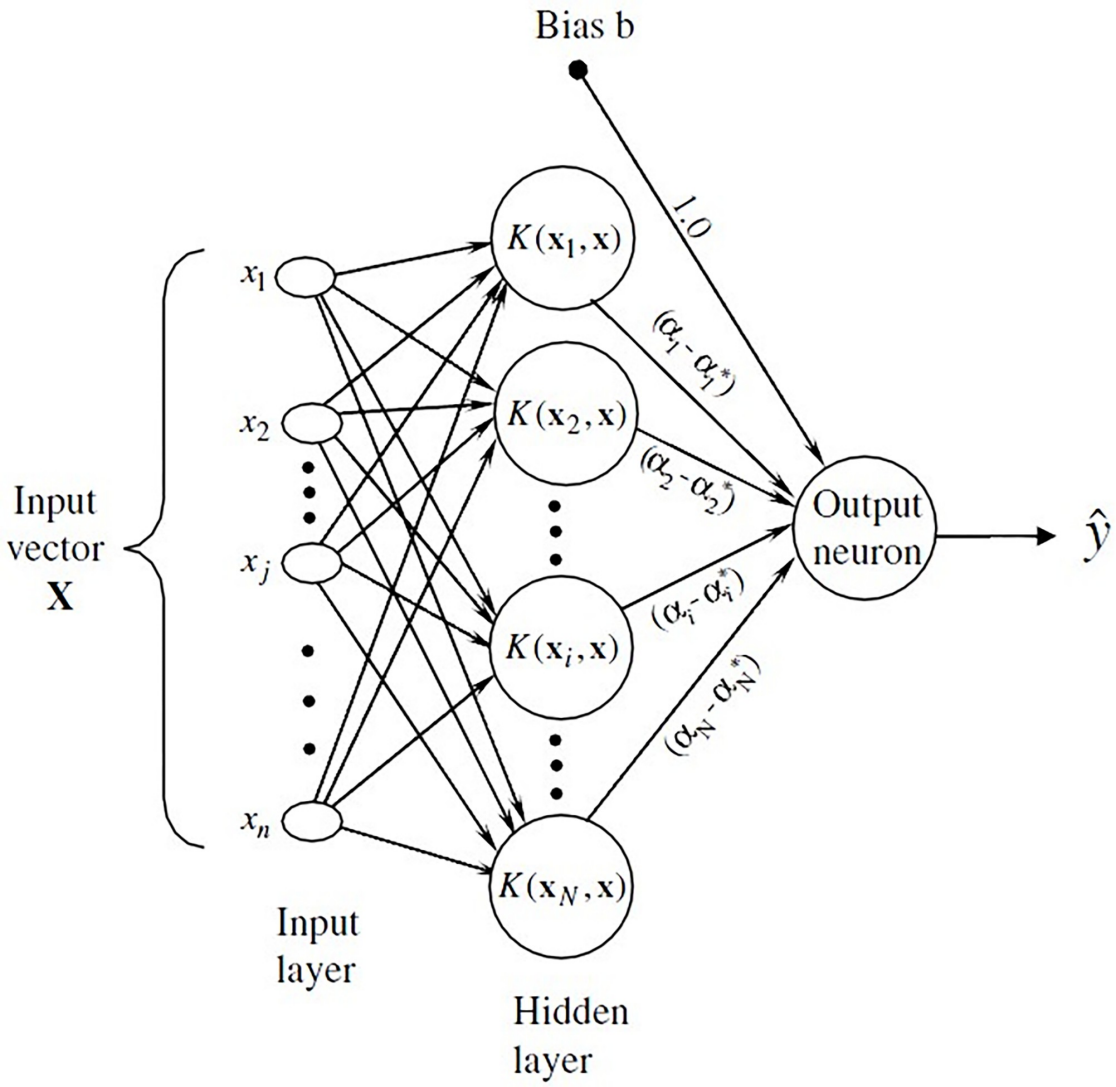
Support Vector Machine (SVM) structure for prediction.

Where, *ϕ*: *R*^*d*^→*F* SVM kernel functions are crucial for the SVM classifier’s ability to handle non-linear data. The linear kernel is straightforward, working well with linearly separable data by calculating the separation in the original feature space [54]. The polynomial kernel extends this capability to non-linear models by computing the similarity of vectors in a transformed space defined by polynomials of the original variables [55]. The Radial Basis Function (RBF) or Gaussian kernel is particularly popular for its ability to map inputs into an infinite-dimensional space, thus facilitating the handling of more complex data structures [56]. Lastly, the sigmoid kernel, akin to a two-layer perceptron neural network, serves as an activation function, transforming the data into a format suitable for classification. Each kernel has distinct parameters that require optimization to achieve the best results, and the choice of kernel significantly influences the SVM’s performance, especially in complex datasets where the relationships between variables are not linear [57]. In this research, based on the data and relation between them, RBF kernel was applied. Fig 1 shows the structure of a typical SVM model.

### 2.19. Radial Basis Function (RBF)

The RBF model is a popular kernel used within SVM frameworks, particularly for non-linear data. It transforms the feature space in such a way that non-linear relationships become linearly separable in the higher-dimensional space [58]. The RBF network is a widely utilized approach for predictive modelling, characterized by its tri-layered structure [59]. The initial layer, or the input layer, corresponds in size to the model’s input count. Each input is weighted, signifying its relative importance [60]. The intermediary layer, or the hidden layer, contains a series of neurons whose quantity is critical for the ANNs efficacy. The final layer, or the output layer, mirrors the desired output dimensionality. In the context of predicting greenhouse temperatures, the output layer is designed with a singular neuron to reflect the singular focus of the study [61]. Distinctively, the RBF network’s hidden layer neurons are governed by a non-linear activation function. The network’s convergence towards the global minimum during the training phase is facilitated by adjusting the bias term. RBF model can be define as [62]:

$$Y=W^{T}\Phi =\sum_{j=1}^{L_{2}}w_{ij}\phi (\|x-c_{i}\|)$$

**Fig 2:**
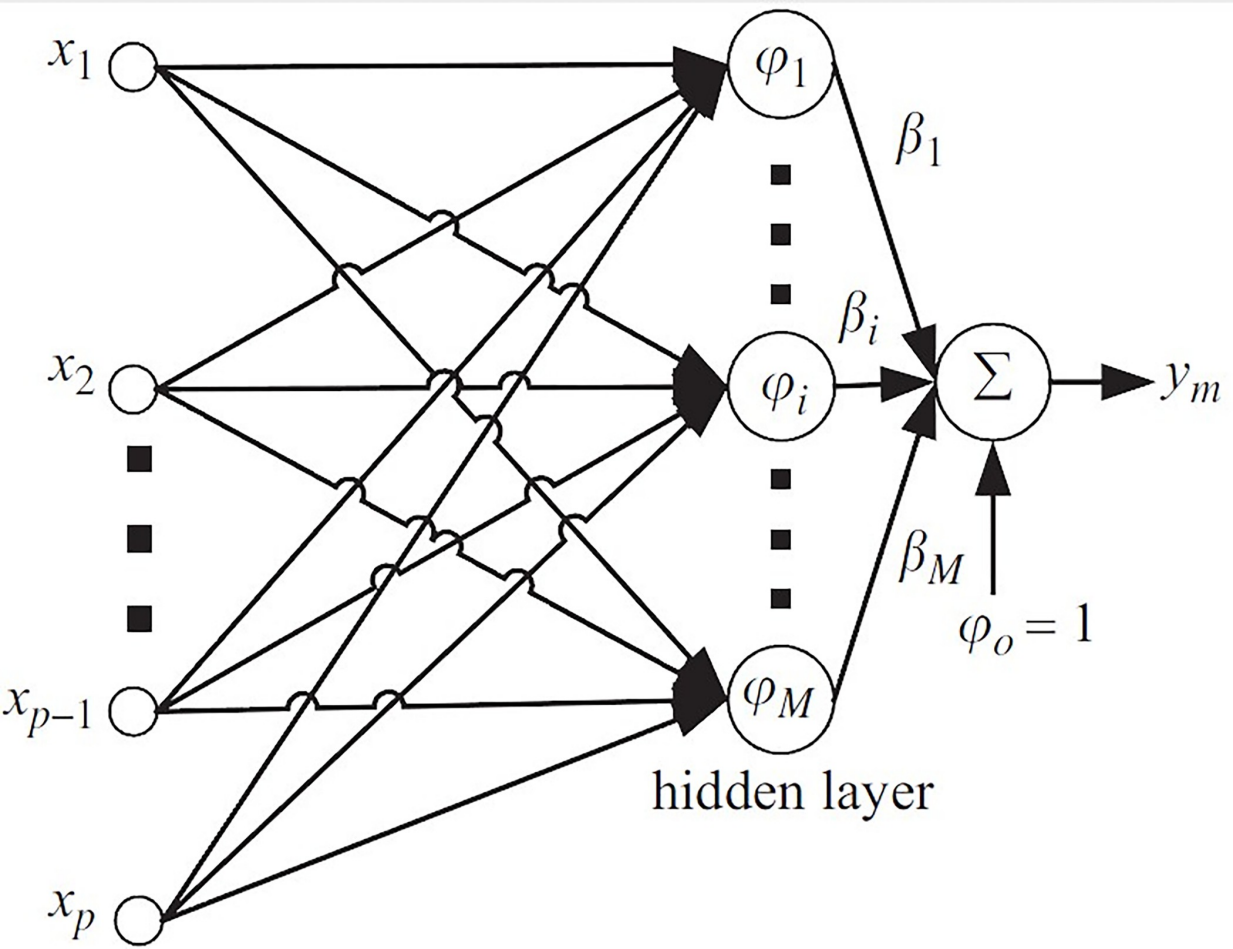
Radial Basis Function (RBF) structure.

In the framework of RBF networks, the response for a given input (x) is computed through the weighted sum of the hidden to output layer connections, symbolized by *w*_*ij*_. The hidden layer’s neuron count is indicated by L_2_ and *c*_*i*_ denotes the centroid of each neuron within this layer. This architecture allows the network to process inputs via a series of transformations, ultimately leading to the desired output through a combination of these weighted connections. In the current investigation, the optimal network architecture was determined by experimenting with the spread parameter, which was adjusted between 0.1 and 1.00 [62]. Various training methodologies are available for fine-tuning a network’s weights to reduce error within the RBF framework. The error backpropagation approach is frequently utilized for this purpose. Specifically, the Bayesian Regularization Backpropagation, denoted as Trainbr, was employed as the training algorithm for the RBF model. Trainbr utilizes Levenberg-Marquardt optimization to refine the network’s weights and biases, aiming to find the ideal balance between minimizing squared errors and weight values to achieve a model with strong generalization capabilities [63]. This technique, known as Bayesian regularization, is particularly advantageous for its capacity to calibrate weights based on their importance in output prediction, thus averting the risk of overfitting. This attribute is especially valuable when working with limited datasets and striving to maximize the size of the training subset. The Trainbr algorithm iteratively optimizes the network, seeking the best error-weight trade-off, resulting in a model that is effective on both training and novel data. In the realm of ANN models, it is possible to emulate any desired continuous function by integrating a hidden layer populated with an adequate count of neurons. For this study, a singular hidden layer was utilized to construct the RBF model [64]. The RBF method’s efficacy in predicting greenhouse temperatures was assessed by varying the neuron count in the hidden layer from 3 to 35, with the aim of identifying the most effective configuration. Consistent with prior studies, linear transfer functions were implemented in the output layer of the RBF method, as they have been shown to proficiently approximate intricate functions. Fig 2 shows the structure of an RBF model.

RBF and SVM models applied in this research for predicting some of food properties including pH, moisture content, total viable count, fungal count, psychrotrophic bacteria count, coliform count, color change (ΔE), hardness and overall acceptance.

To assess the performance of both Gaussian Process Regression and Multilayer Perceptron models, various statistical metrics were employed, including the Root Mean Square Error (RMSE), Mean Absolute Percentage Error (MAPE) and the coefficient of determination R^2^ [62]:

$$MAPE=\frac{1}{n}\sum_{j=1}^{n}\left|\frac{T_{d_{j}}-T_{p_{j}}}{T_{d_{j}}}\right|\times 100$$

$$R^{2}={\left[\frac{\sum_{j=1}^{n}(T_{d_{j}}-\bar{Td})(T_{p_{j}}-\bar{Tp})}{\sum_{j=1}^{n}(T_{d_{j}}-\bar{Td})\times \sum_{j=1}^{n}(T_{p_{j}}-\bar{Tp})}\right]}^{2}$$

Where n is the experimental data, Tp_j_ is the predicted data by the models, Td_j_ is the actual data and $\bar{Td}$ and $\bar{Tp}$ are the average values of actual and predicted data.

## 3. Results and discussion

### 3.1. Propolis extract characterization

#### 3.1.1. Phenolic compounds

The PE composition contained a variety of important compounds at different concentrations (Table 1). The extract included gallic acid (98.593), benzoic acid (119.972), syringic acid (91.654), 4–3 dimethoxy cinnamic acid (323.534), *p*-coumaric acid (492.698), myricetin (90.277), caffeic acid (159.184), luteolin (143.543), chlorogenic acid (105.333), and apigenin (100.431), all of which are recognized for their potential health benefits and biological activities. These findings are consistent with Kasiotis et al. (2017) [35] research. Gallic acid is a phenolic compound known for its antioxidant properties [65], while benzoic acid is commonly used as a preservative in food products [66]. Syringic acid has been investigated for its anti-inflammatory and antioxidant effects [67]. Additionally, 4–3 dimethoxy cinnamic acid, *p*-coumaric acid, and caffeic acid are phenolic acids with diverse biological activities [68]. Luteolin and apigenin are flavonoids recognized for their anti-inflammatory and antioxidant properties, and myricetin is another flavonoid with potential health benefits [69]. Chlorogenic acid, found in coffee and other plant sources, has been studied for its antioxidant and anti-inflammatory effects [70]. Further research and analysis may be necessary to fully comprehend the combined effects of these compounds in PE and their implications for health and well-being.

**Table 1:** The composition of propolis extract.

No.	Compound	Concentration (mg/g)	Retention time (min)
1	Gallic acid	98.593	3.61
2	Benzoic acid	119.972	4.09
3	Syringic acid	91.654	4.91
4	4–3, dimethoxy cinnamic acid	323.534	7.35
5	*p*-coumaric acid	492.698	10.24
6	Myricetin	90.277	11.59
7	Caffeic acid	159.184	14.19
8	Luteolin	143.543	14.47
9	Chlorogenic acid	105.333	15.21
10	Apigenin	100.431	16.29

The PE was determined to have a TPC of 36.67 ± 0.57 mg GAE/g and a TFC of 48.02 ± 0.65 mg QE/g. It is important to note that the TPC and TFC values of PE can vary based on the source and processing methods used. For instance, phenolic Mediterranean propolis has been reported to have a TPC ranging from 14.0 to 189.7 mg GAE/g, while the total flavonoid content ranges between 7.2 and 103.9 mg QE/g [71]. Chinese poplar-type propolis has shown TPC values ranging from 87.11 to 257.93 mg GAE/g and TFC values ranging from 105.25 to 351.25 mg QE/g [72]. Croatian propolis has been found to have a TPC range of 70–220 mg GAE/g [73]. In Anatolian propolis, derived from *Populus nigra*, *P*. *tremula*, and non-poplar types, phenolic and flavonoid content varies from 11.24 to 47.15 mg GAE/g and 3.88 to 48.70 mg QE/g, respectively [74]. Overall, propolis is recognized for its high levels of phenolic compounds and flavonoids, which contribute to its antioxidant properties.

#### 3.1.2. FTIR

**Fig 3:**
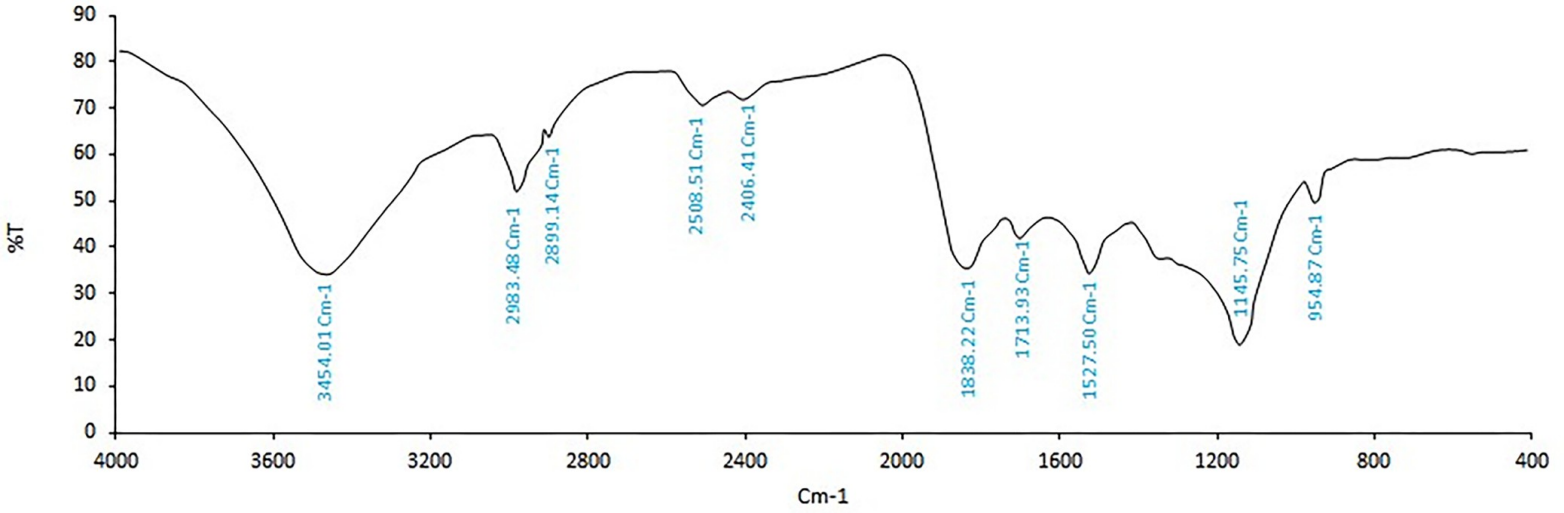
Fourier transform infrared (FTIR) spectrum of propolis extract.

The provided FTIR spectrum of PE reveals the presence of various functional groups in the sample (Fig 3). The propolis sample showed a strong band at 3454 cm^−1^, which could be attributed to the O–H stretching vibration of the phenolic group [71]. Additionally, the peaks at 2983 cm^−1^ and 2899 cm^−1^ indicate the presence of C-H alkane groups. Moreover, the peaks at 2508 cm^−1^ and 2406 cm^−1^ could suggest the existence of C-H hydrocarbons [75]. The absorption at 1838 cm^−1^ pointed to the presence of carbonyl compounds, while the peak at 1713 cm^−1^ could be due to the carbonyl group (C = O) stretching vibrations of the ester bond, possibly indicating the presence of monoesters from beeswax in propolis. A weak band at 1527 cm^−1^ can be attributed to flavonoids, specifically C = C (aromatic ring) stretching. The broad band observed at 1145 cm^−1^ corresponds to the C–O asymmetric stretching vibration of esters related to long-chain aliphatic acids [71,76].

#### 3.1.3. Antioxidant effect

**Fig 4:**
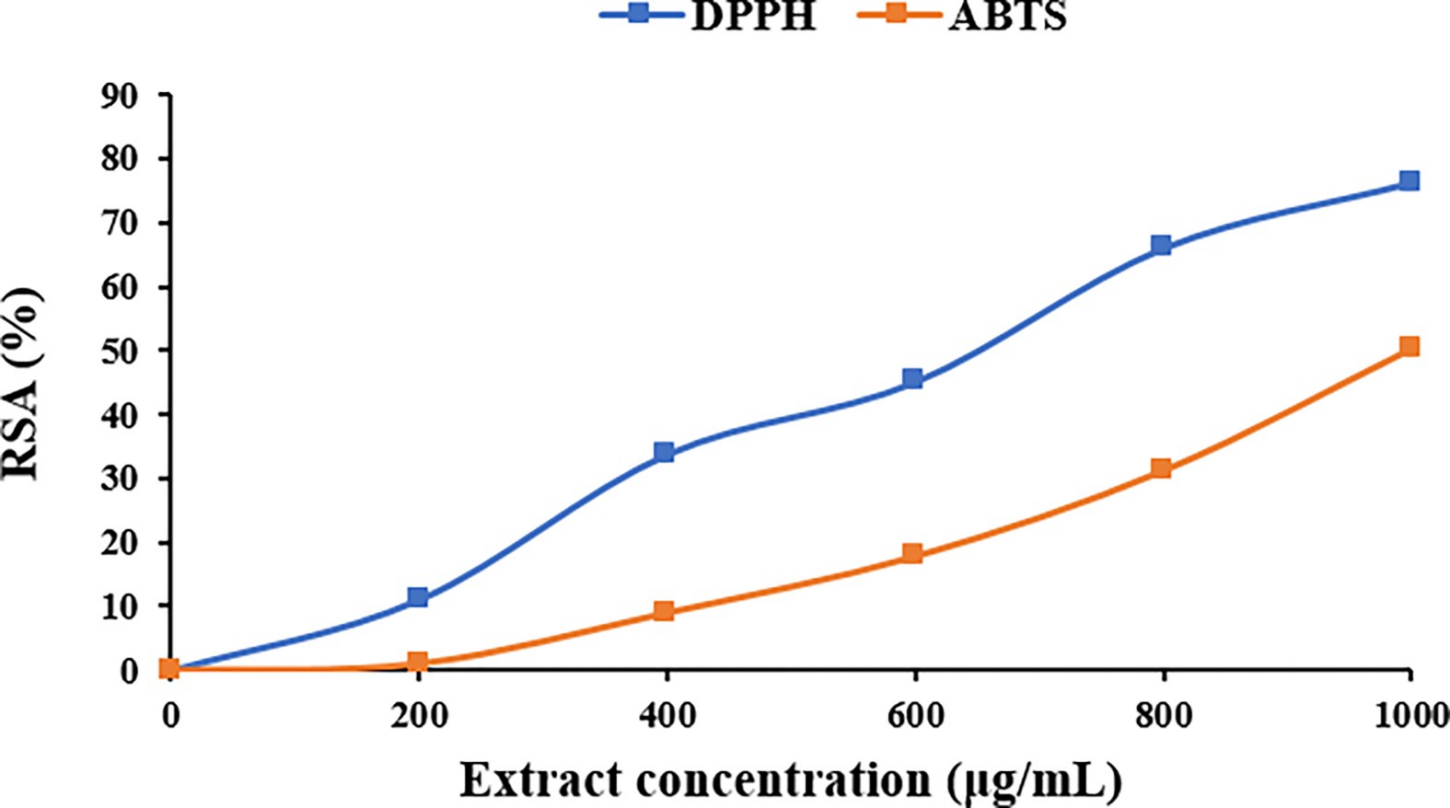
Radical scavenging activity (RSA) of propolis extract (PE) against DPPH and ABTS radicals.

PE is recognized for its potent antioxidant properties attributed to the presence of phenolic compounds and flavonoids. These active components play a role in counteracting harmful free radicals in the body, which can lead to oxidative stress and cell damage. The antioxidant activity of PE was dose-dependent, as evidenced by the marked increase in radical scavenging activity with higher extraction concentrations (Fig 4). The extract’s radical scavenging activity ranged from 0 to 76.22% for DPPH radicals and from 0 to 50.31% for ABTS radicals. It’s important to note that the antioxidant effectiveness of PE can vary based on factors such as propolis source, processing methods, and concentration of bioactive compounds. Studies have shown that Brazilian PEs exhibit antioxidant activity within the range of 4.3–87.5% using the DPPH method [77]. Additionally, the antioxidant activity of ethanolic PEs was found to be ranged from 65–160 μg ml^-1^ based on the DPPH radical scavenging method [78]. Laskar et al. (2010) [79] reported that the IC_50_ value of propolis aqueous extract was 0.05 mg/mL, whereas the IC_50_ value of propolis ethanolic extract was 0.07 mg/mL. Thus, propolis aqueous extract exhibited higher radical scavenging activity compared to propolis ethanolic extract, possibly due to its higher polyphenol content and better solubility of polyphenol constituents in water. In general, PE is considered a valuable natural antioxidant that may provide various health benefits.

#### 3.1.4. Antimicrobial effect

Tables 2 and 3 present the antimicrobial impact of PE on various bacterial species at different concentrations. The findings indicate that the antibacterial effect of PE varies significantly based on both the bacterial species and the concentration of the extract. The data demonstrated that the PE exhibited a concentration-dependent antibacterial effect against the tested bacterial species. Furthermore, there was a direct correlation between the concentration of the PE and the size of the inhibition zone (p < 0.05), indicating a stronger antibacterial effect at higher concentrations. While there were no significant differences between bacterial types.

**Table 2:** Antimicrobial effect of propolis extract based on disc diffusion agar method.

Bacterial species	Extract concentration (mg/mL)					Chloramphenicol
	32	64	128	256	512	
*L*. *innocua*	6.20 ± 0.04 ^i^	6.60 ± 0.10 ^hi^	7.70 ± 0.18 ^fgh^	9.50 ± 0.15 ^de^	12.90 ± 0.41 ^a^	26.00 ± 0.47
*S*. *aureus*	6.00 ± 0.00 ^i^	6.20 ± 0.02 ^i^	7.70 ± 0.44 ^fgh^	9.80 ± 0.50 ^cd^	12.60 ± 0.11 ^ab^	23.00 ± 0.52
*E*. *coli*	6.00 ± 0.00 ^i^	6.70 ± 0.20 ^hi^	7.80 ± 0.33 ^fgh^	9.10 ± 0.29 ^def^	11.20 ± 0.20 ^bc^	25.00 ± 0.33
*S*. *typhi*	6.00 ± 0.00 ^i^	6.70 ± 0.25 ^hi^	8.30 ± 0.21 ^efg^	10.10 ± 0.41 ^cd^	11.70 ± 0.25 ^ab^	24.00 ± 0.49
*P*. *aeruginosa*	6.00 ± 0.00 ^i^	6.20 ± 0.13 ^i^	7.40 ± 0.47 ^ghi^	9.50 ± 0.18 ^de^	11.70 ± 0.23 ^ab^	23.00 ± 0.50
*B*. *cereus*	6.10 ± 0.00 ^i^	6.90 ± 0.12 ^ghi^	8.00 ± 0.13 ^fgh^	10.00 ± 0.13 ^cd^	12.20 ± 0.43 ^ab^	25.00 ± 0.41

The means that have different superscript letters show a significant difference (p < 0.05).

**Table 3:** Antimicrobial effect of propolis extract based on well diffusion agar method.

Bacterial species	Extract concentration (mg/mL)				
	32	64	128	256	512
*L*. *innocua*	6.50 ± 0.14 ^hi^	7.20 ± 0.21 ^fghi^	9.40 ± 0.35 ^efghi^	11.30 ± 0.45 ^bcdefgh^	15.50 ± 0.71 ^ab^
*S*. *aureus*	6.00 ± 0.12 ^i^	6.70 ± 0.12 ^hi^	8.80 ± 0.14 ^fghi^	11.30 ± 0.61 ^bcdefgh^	15.20 ± 0.31 ^abc^
*E*. *coli*	6.20 ± 0.10 ^i^	6.80 ± 0.15 ^ghi^	8.10 ± 0.13 ^fghi^	10.30 ± 1.29 ^cdefghi^	14.60 ± 0.21 ^abcd^
*S*. *typhi*	6.60 ± 0.12 ^hi^	7.90 ± 0.12 ^fghi^	9.20 ± 0.25 ^efghi^	13.90 ± 0.11 ^abcde^	15.00 ± 0.66 ^abc^
*P*. *aeruginosa*	6.00 ± 0.11 ^i^	6.70 ± 0.23 ^hi^	8.90 ± 0.27 ^fghi^	11.70 ± 0.78 ^bcdef^	18.50 ± 1.51 ^a^
*B*. *cereus*	6.50 ± 0.10 ^hi^	7.40 ± 0.95 ^fghi^	9.80 ± 1.83 ^defghi^	12.00 ± 2.20 ^bcdef^	18.00 ± 2.40 ^a^

The means that have different superscript letters show a significant difference (p < 0.05).

Table 4 outlines the antimicrobial effect of PE based on MIC and MBC methods. The MIC values represent the minimum concentration of propolis extract required to inhibit bacterial growth, while the MBC values indicate the minimum concentration required to kill the bacteria. Notably, the MIC for *B*. *cereus* was 16 mg/mL, the lowest among the listed bacteria, suggesting its high sensitivity to PE. The MBC is 256 mg/mL. Overall, Gram-positive bacteria exhibited greater sensitivity to the extract compared to Gram-negative bacteria. This data offers quantitative data on the efficacy of PE against different bacteria, which is essential for evaluating its potential use as an antibacterial agent.

**Table 4:** Antimicrobial effect of propolis extract based on minimum inhibitory concentration (MIC) and minimum bactericidal concentration (MBC) methods.

Bacterial species	MIC (mg/mL)	MBC (mg/mL)
*L*. *innocua*	32	256
*S*. *aureus*	32	512
*E*. *coli*	64	> 512
*S*. *typhi*	64	> 512
*P*. *aeruginosa*	64	> 512
*B*. *cereus*	16	256

According to some researchers, the biological effects of Brazilian propolis are primarily attributed to the high levels of phenolic acids, while flavonoids are believed to be responsible for the activity of European propolis extracts [77]. Studies have shown that propolis demonstrates a wide range of antibacterial activities, with greater efficacy against Gram-positive bacteria than Gram-negative bacteria [80]. For example, propolis has exhibited strong synergistic effects when combined with honey against antibiotic-resistant *S*. *aureus* and *E*. *coli* [81]. Additionally, research on red propolis has highlighted its potential as a source of antimicrobial phytochemicals. Utilizing an alcohol-free high-performance extraction method, researchers were able to recover antibacterial and antioxidant phytochemicals from red propolis. The extract demonstrated inhibitory effects on the growth of *S*. *aureus* and *Salmonella enteritidis* bacteria [82]. Furthermore, the antibacterial activity of propolis can vary based on its geographical origin. For instance, propolis from the Middle East exhibited high activity against both Gram-positive (*S*. *aureus*) and Gram-negative (*E*. *coli*) strains, whereas propolis samples from Germany, Ireland, and Korea showed lower activity [80]. In summary, propolis shows promise as a natural substance with significant antibacterial properties. However, further research is necessary to fully comprehend its potential and to standardize its application in various contexts.

**Fig 5:**
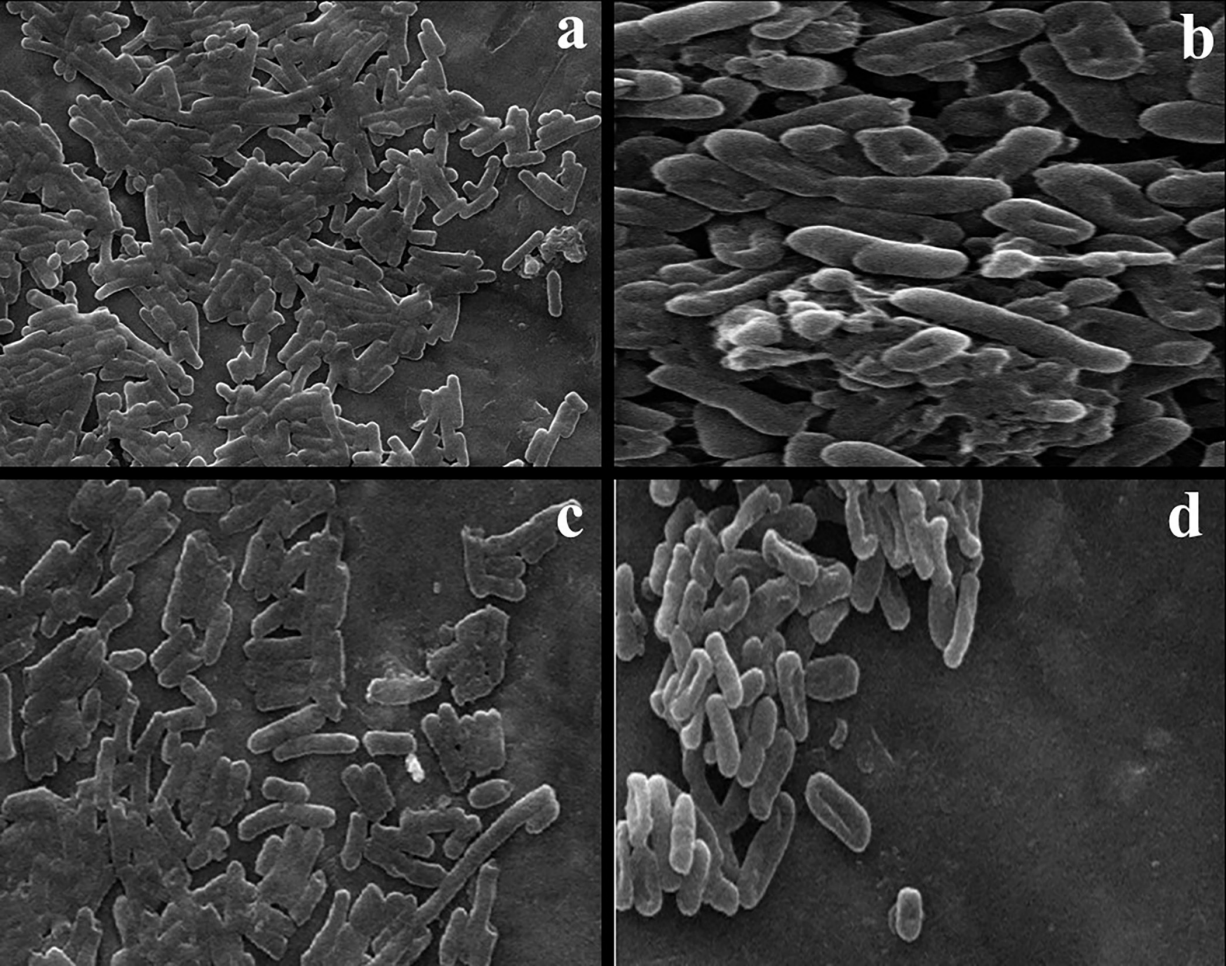
Scanning electron microscopy (SEM) images of non-treated *Escherichia coli* (a), treated *E*. *coli* (b), non-treated *Salmonella typhi* (c), and treated *S*. *typhi* (d) with propolis extract (PE).

Antimicrobial compounds can often eliminate microorganisms by modifying their structures and affecting their vital components. When untreated strains of *E*. *coli* and *S*. *typhi* were examined under SEM, their rod structures were found to be distorted (Fig 5A and 5C). In contrast, the treated strain displayed cell walls that were damaged, torn, and punctured (Fig 5B and 5D). The extract’s effect on the cells led to heightened permeability, membrane ruptures, and the release of cytoplasmic contents, ultimately causing cell death.

The antibacterial properties of propolis can be viewed from two perspectives. Firstly, it directly affects microorganisms, and secondly, it stimulates the immune system, activating the body’s natural defenses [83]. Studies on propolis mechanisms suggest that it influences the permeability of microbial cell membranes, disrupts membrane potential, reduces adenosine triphosphate (ATP) production, and inhibits bacterial mobility [80]. Artepillin C (3,5-diprenyl-*p*-coumaric acid), a phenolic compound found in propolis, has demonstrated antibacterial effects against *S*. *aureus* [84]. Additionally, an ethanolic PE with high concentrations of kaempferide, artepillin-C, drupanin, and *p*-coumaric acid has shown antioxidant and antibacterial properties against *S*. *aureus*, *Staphylococcus saprophyticus*, *Listeria monocytogenes*, and *Enterococcus faecalis* [85]. Furthermore, flavonoids like pinocembrin and apigenin have demonstrated antibacterial activity against *Streptococcus mutans* [86], while isolated apigenin has been effective against Gram-negative bacteria such as *P*. *aeruginosa*, *Klebsiella pneumoniae*, *Salmonella enterica* serotype Typhimurium, *Proteus mirabilis*, and *Enterobacter aerogenes* [87]. Cinnamic acid and its derivatives have also been found to possess antimicrobial properties against both Gram-positive and Gram-negative microorganisms, through anti-quorum sensing activity and damage to the cell membrane, leading to inhibition of ATPases, cell division, and biofilm formation [88].

#### 3.1.5. Cytotoxic effect

**Fig 6:**
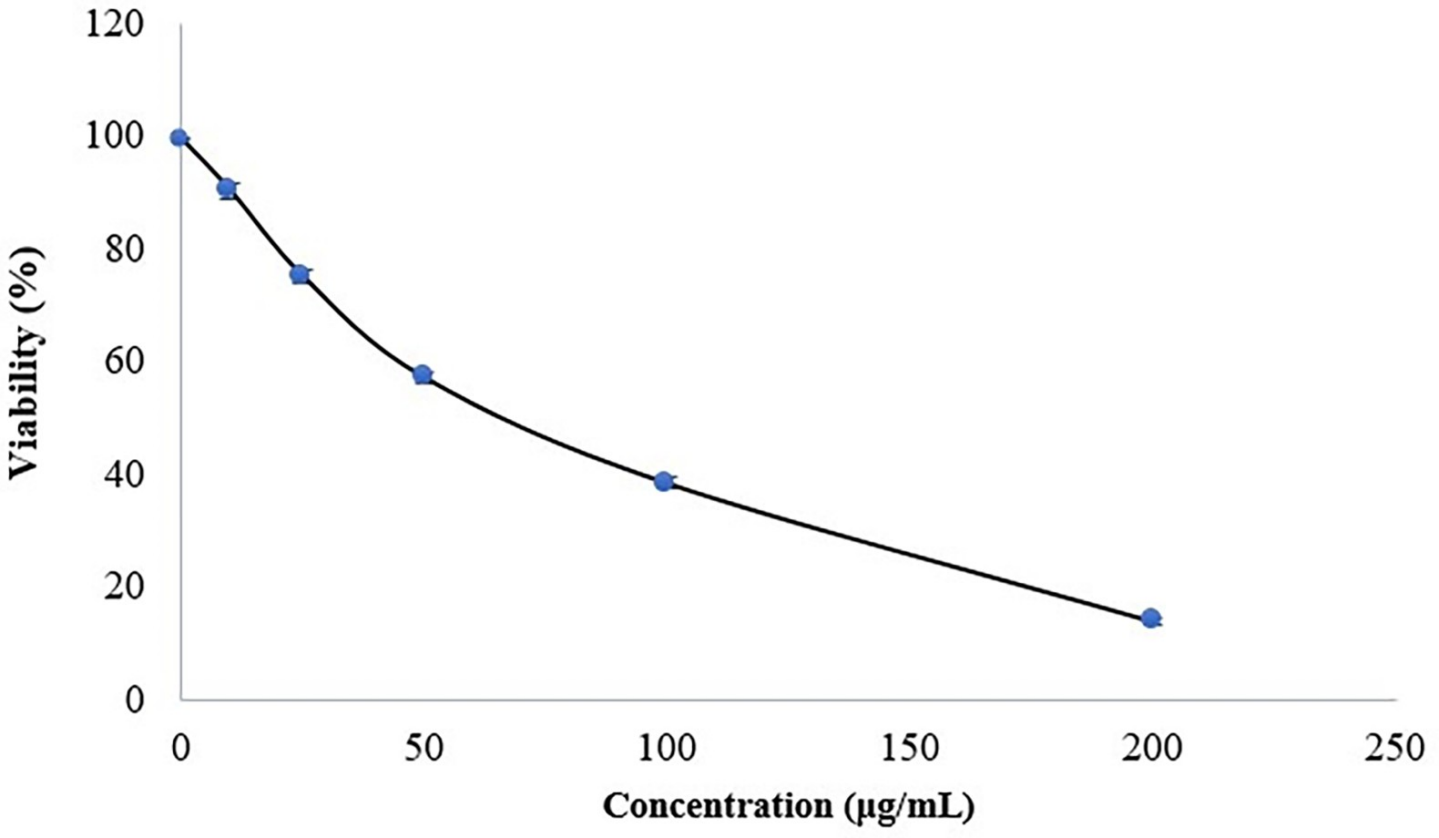
Cytotoxic effect of various concentrations of propolis extract on survival of C115 HeLa cell line.

The PE was also studied for its potential cytotoxic effects on various types of cells, including human cervical cancer cells. According to the findings shown in Fig 6, the viability of C115 HeLa cells was observed to be 99.34%, 90.24%, 75.01%, 57.12%, 38.41%, and 139.93% for propolis concentrations of 0, 10, 25, 50, 100, and 200 μg/mL, respectively. The IC_50_ value was determined to be 94.14 μg/mL. These results clearly demonstrate a significant decrease in C115 HeLa cell viability as the concentration of propolis increased. The PE may, therefore, exhibit cytotoxicity against certain cancer cell lines, potentially due to its bioactive compounds such as flavonoids and phenolic acids.

The literature has reported the cytotoxic effect of PE on cancer cells [89–91]. The ethanol extract of propolis from *Tetragonisca fiebrigi* has also demonstrated cytotoxic activity, which has been attributed to the presence of phenolic compounds like coumaric acid, cinnamic acid, and derivatives [89]. These compounds have exhibited cytotoxic action against leukemia, human cervical adenocarcinoma, and human prostate cancer cell lines [92–94]. However, recent studies suggest that the cytotoxic and anti-proliferative effects of propolis on tumor cells may not be solely dependent on the concentration of a specific component, but instead may rely on the synergism between actions of several components [95,96].

### 3.2. Biological properties of *Lepidium sativum* mucilage

The LS was determined to have a TPC of 15.23 ± 0.43 mg GAE/g and a TFC of 11.51± 0.61 mg QE/g (Table 5). The LS was determined to have a DPPH of 429.65 ± 1.28 μg/mL and a ABTS of 403.59 ± 1.46 μg/mL (Table 5). The antioxidant properties of LS are directly influenced by its phenolic and flavonoid compounds. The number of hydroxyl groups in antioxidants does not solely determine their effectiveness. Factors such as the positioning of hydroxyl groups, presence of other functional groups like double bonds, and the combination of hydroxyl and ketone groups are crucial for antioxidant activity. Variations in the results of antioxidant activity and phenolic compounds in LS across different studies could be attributed to factors such as climate, drying methods, and extraction techniques. Research has demonstrated that a high TPC is a key factor in the strong antioxidant abilities of certain mucilage and extracts, as there is a direct correlation between TPC and antioxidant activity in plants [38,40].

**Table 5:** Biological properties of *Lepidium sativum* mucilage (LS).

LS	Result
TPC	15.23 ± 0.43 mg GAE/g
TFC	11.15 ± 0.61 mg QE/g
DPPH (IC_50_)	429.65 ± 1.28 μg/mL
ABTS (IC_50_)	403.59 ± 1.46 μg/mL

The results of the effect of LS on pathogenic bacteria in the Table 6 is brought. The results showed that LS had a greater effect on Gram-positive bacteria compared to Gram-negative bacteria. Alizadeh Behbahani and Imani Fooladi (2018) [38] conducted a study on the antibacterial properties of *Lallemantia royleana* seed mucilage against various pathogens such as *P*. *aeruginosa*, *E*. *coli*, *B*. *cereus*, *S*. *aureus*, *Streptococcus pyogenes*, *Bacillus subtilis*, and *Candida albicans* “*in vitro*”. Their findings revealed that 2 mg/mL of *Lallemantia royleana* seed mucilage effectively inhibited the growth of *B*. *subtilis* and *S*. *pyogenes*. However, this same concentration had no significant impact on *B*. *cereus*, *E*. *coli*, and *P*. *aeruginosa*, indicating that it was not effective in preventing the growth of these bacteria in culture. One potential explanation for the varying antimicrobial effects of extract or mucilage on Gram-positive bacteria compared to Gram-negative bacteria lies in the disparities of their cell wall compositions. Gram-positive bacteria possess a thicker peptidoglycan layer in their cell wall, allowing antimicrobial agents to more easily penetrate and disrupt their cellular structure [39,97–99]. On the other hand, Gram-negative bacteria feature an additional outer membrane that serves as a protective barrier, hindering the access of antimicrobial compounds to their target sites. Furthermore, Gram-negative bacteria may possess efflux pumps that actively expel antimicrobial substances from the cell, diminishing their efficacy. These factors collectively contribute to the differential antimicrobial responses observed in Gram-positive and Gram-negative bacteria [100–102].

**Table 6:** Antimicrobial effects of *Lepidium sativum* mucilage (LS) on some pathogenic bacteria (pour plate method).

Bacterial species	Result^a^
*L*. *innocua*	S
*S*. *aureus*	S
*E*. *coli*	R
*S*. *typhi*	I
*P*. *aeruginosa*	I
*B*. *cereus*	S

^a^R, resistant; I, intermediate; S, sensitive.

### 3.3. Bio-preservation of raw buffalo meat

#### 3.3.1. Microbial changes

Table 7 presents microbial status of buffalo meat as a function of storage time and edible coating type. Overall, as the storage time increased, the total bacterial count generally increased in all samples (p < 0.05). However, the samples treated with PE exhibited lower bacterial counts compared to the control group and the LS treated sample, indicating a potential inhibitory effect of PE on bacterial growth. This effect was more noticeable at higher PE concentrations. For instance, the control group had the highest bacterial count (8.00 log CFU/g), while the LS+2.5%PE sample had the lowest (6.23 log CFU/g). This suggests that PE effectively reduces bacterial growth, particularly at higher concentrations and over longer storage durations.

**Table 7:** Microbial counts of buffalo meat as a function of storage time and edible coating type.

Parameters	Time (days)	Samples				
		Control	LS	LS+0.5%PE	LS+1.5%PE	LS+2.5%PE
Total viable count (log CFU/g)	1	4.83 ± 0.27 fg	4.62 ± 0.24 fg	4.50 ± 0.16 fg	4.35 ± 0.27 fg	4.10 ± 0.20 g
	3	7.12 ± 0.34 cde	6.75 ± 0.29 cde	6.00 ± 0.17 def	5.85 ± 0.30 defg	5.70 ± 0.38 efg
	6	9.25 ± 0.44 ab	8.10 ± 0.38 bc	7.68 ± 0.39 bcd	7.40 ± 0.25 bcde	7.10 ± 0.36 cde
	9	10.80 ± 0.46 a	9.20 ± 0.37 ab	8.40 ± 0.47 bc	8.20 ± 0.34 bc	8.00 ± 0.24 bc
					
Psychrotrophic bacteria count (log CFU/g)	1	2.70 ± 0.15 ghi	2.55 ± 0.20 hi	2.30 ± 0.19 i	2.25 ± 0.11 i	2.20 ± 0.11 i
	3	3.90 ± 0.32 efg	3.70 ± 0.19 fgh	2.80 ± 0.13 ghi	2.70 ± 0.15 ghi	2.65 ± 0.17 hi
	6	5.70 ± 0.18 abcd	5.00 ± 0.23 bcde	5.00 ± 0.18 bcde	4.80 ± 0.29 cdef	4.50 ± 0.19 def
	9	6.60 ± 0.28 a	6.20 ± 0.26 ab	5.90 ± 0.31 abc	5.75 ± 0.24 abc	5.50 ± 0.20 abcd
					
Coliforms (log CFU/g)	1	2.50 ± 0.14 def	2.30 ± 0.19 ef	2.30 ± 0.17 ef	2.30 ± 0.11 ef	2.10 ± 0.11 f
	3	3.30 ± 0.16 bcde	3.10 ± 0.12 bcdef	3.00 ± 0.15 cdef	2.50 ± 0.21 def	2.35 ± 0.20 ef
	6	4.20 ± 0.19 ab	3.70 ± 0.14 abc	3.40 ± 0.24 bcde	3.21 ± 0.18 bcde	3.11 ± 0.27 bcdef
	9	4.80 ± 0.25 a	4.20 ± 0.23 ab	3.90 ± 0.21 abc	3.60 ± 0.24 bcd	3.55 ± 0.25 bcd
					
Fungi (log CFU/g)	1	2.50 ± 0.19 fgh	2.35 ± 0.20 fgh	2.20 ± 0.11 gh	2.15 ± 0.10 gh	2.00 ± 0.10 h
	3	3.10 ± 0.25 defgh	2.60 ± 0.27 efgh	2.42 ± 0.21 fgh	2.30 ± 0.15 fgh	2.10 ± 0.15 gh
	6	4.80 ± 0.20 ab	3.70 ± 0.17 bcde	3.40 ± 0.18 cdef	2.30 ± 0.17 fgh	2.25 ± 0.20 gh
	9	5.30 ± 0.32 a	4.30 ± 0.23 abc	3.80 ± 0.19 bcd	3.40 ± 0.20 cdef	3.20 ± 0.17 cdefg

LS–*Lepidium sativum* mucilage; PE–propolis extract.

The means that have different superscript letters show a significant difference (p < 0.05).

Psychrotrophic bacteria are capable of growing and multiplying at refrigeration temperatures, which can cause spoilage of meat products. Therefore, it is important to monitor and control the psychrotrophic bacteria count in meat to ensure its quality and safety. The data in Table 7 shows that the psychrotrophic bacteria count of buffalo meat varies significantly depending on the storage time and the type of edible coating used. The psychrotrophic bacteria count of the samples increased significantly from 2.40 to 5.99 log CFU/g as the storage time increased from 1 to 9 days (p < 0.05). Certain types of edible coatings, particularly LS+2.5%PE, may have a more significant impact on reducing psychrotrophic bacteria count compared to others. At the end of the storage period, the LS+2.5%PE had remarkably lower psychrotrophic bacteria count compared to the control (5.50 vs. 6.60 log CFU/g). The psychrotrophic bacteria count decreased from 4.73 log CFU/g in the control to 3.71 log CFU/g in the LS+2.5%PE as the PE content increased in the edible coating (p < 0.05). Samples with lower psychrotrophic bacteria count may indicate better antimicrobial properties of the edible coating used. This information is valuable for determining which coating types are most effective in preserving the quality and safety of buffalo meat over an extended storage period.

The coliform levels in buffalo meat coated with different edible coatings were assessed at various storage durations (Table 7). The findings indicated significant variations in coliform levels across the different coatings and storage periods. Generally, coliform counts were higher in the control group and decreased as the concentration of PE in the coatings increased. Notably, the LS+2.5%PE and control samples exhibited the lowest (2.78 log CFU/g) and highest (3.70 log CFU/g) coliform counts, respectively (p < 0.05). Furthermore, coliform counts were observed to be higher during longer storage periods (p < 0.05). The results suggest that edible coatings with higher PE concentrations effectively reduced coliform counts in buffalo meat during storage.

The number of fungi increased with the duration of storage for all coatings (p < 0.05) (Table 7). However, there were notable differences in fungi counts among the various coatings. The control group exhibited the highest fungi counts at all storage times (3.93 log CFU/g), while the LS+2.5%PE sample had the lowest fungi counts (2.39 log CFU/g). These findings indicate that incorporating high levels of PE into edible coatings can effectively inhibit fungi growth in buffalo meat.

The use of bioactive edible coatings can significantly impact the microbial, physical, and sensory characteristics of buffalo meat. The higher shelf life of buffalo meats coated with PE-loaded edible coatings could be due to the antimicrobial effect of PE, as indicated in Tables 2–4. Saffari Samani’s study revealed that applying *Zataria multiflora* Boiss essential oil-loaded Zedo gum edible coating to buffalo samples had a strong antimicrobial effect on various microorganisms, including total viable count and psychrotrophic bacteria count, *E*. *coli*, coliforms, *S*. *aureus*, and fungi [5]. Edible films and coatings are primarily composed of proteins, lipids, or polysaccharides, forming a structural matrix that acts as a physical barrier against spoilage microorganisms. Additionally, the incorporation of phenolic compounds can enhance the bioactive potential of these polymeric matrices, as phenolic compounds are known for their antioxidant and antimicrobial properties. These active coatings play a role in controlling moisture transfer, gas exchange, microbial growth, oxidation processes, and other chemical reactions, thereby helping to prevent food spoilage and extend shelf life [103,104].

#### 3.3.2. Physical properties

The moisture content of buffalo meat decreased with increasing storage time (p < 0.05) (Table 8). The control group (no coating) consistently had a significantly lower moisture content (69.92%) compared to the other groups throughout the storage period. The LS group showed a significantly higher moisture content than the control group at all storage times. Additionally, the moisture content of the LS group with added 2.5% PE (73.53%) was significantly higher than that of the control group at all storage times. At 1 day, the moisture content of all groups was statistically similar. However, at 3, 6, and 9 days, the moisture content of the LS+2.5% PE group was significantly higher than that of the control group. These findings suggest that adding PE to LS coatings can effectively help retain moisture in buffalo meat during storage. This positive impact of the edible coating may be attributed to its lower permeability to water vapor and its physical barrier function [5]. High moisture retention in meat when applying PE compared to control can be significantly influenced by various factors, including pH changes. The application of PE can alter the protein structure within the buffalo meat. Extracts often contain ingredients that serve as moisture binding agents. As a result, moisture retention in the coated samples containing the PE was better than the control sample over time [5,97].

**Table 8:** Physical properties of buffalo meat as a function of storage time and edible coating type.

Parameters	Time (days)	Samples				
		Control	LS	LS+0.5%PE	LS+1.5%PE	LS+2.5%PE
Moisture content (%)	1	73.37 ± 0.66 abcd	73.70 ± 0.32 abc	73.90 ± 0.34 ab	74.10 ± 0.27 a	74.50 ± 0.20 a
	3	70.19 ± 0.45 fg	72.50 ± 0.34 abcde	72.88 ± 0.30 abcde	73.80 ± 0.29 abc	74.00 ± 0.28 a
	6	68.60 ± 0.60 gh	71.42 ± 0.28 def	71.89 ± 0.20 bcdef	72.50 ± 0.20 abcde	73.10 ± 0.16 abcd
	9	67.50 ± 0.50 h	70.40 ± 0.32 fg	70.90 ± 0.24 ef	71.80 ± 0.32 cdef	72.50 ± 0.32 abcde
					
pH	1	5.88 ± 0.05 ab	5.87 ± 0.04 ab	5.80 ± 0.05 ab	5.75 ± 0.06 b	5.73 ± 0.08 b
	3	5.95 ± 0.10 ab	5.90 ± 0.07 ab	5.82 ± 0.09 ab	5.82 ± 0.07 ab	5.80 ± 0.06 ab
	6	6.15 ± 0.11 ab	6.00 ± 0.08 ab	5.89 ± 0.07 ab	5.87 ± 0.09 ab	5.85 ± 0.10 ab
	9	6.20 ± 0.08 a	6.10 ± 0.05 ab	5.92 ± 0.08 ab	5.90 ± 0.10 ab	5.90 ± 0.08 ab
					
Hardness (N)	1	57.90 ± 0.74 abcd	58.50 ± 0.66 abc	59.10 ± 0.49 ab	59.30 ± 0.43 ab	59.60 ± 0.40 a
	3	55.50 ± 0.49 de	57.60 ± 0.48 abcd	58.00 ± 0.67 abcd	58.80 ± 0.38 abc	58.90 ± 0.24 ab
	6	53.49 ± 0.52 ef	56.10 ± 0.48 cde	57.30 ± 0.43 abcd	57.60 ± 0.47 abcd	57.90 ± 0.37 abcd
	9	51.70 ± 0.47 f	53.50 ± 0.38 ef	55.35 ± 0.38 de	56.80 ± 0.54 bcd	57.00 ± 0.42 abcd
					

LS–*Lepidium sativum* mucilage; PE–propolis extract.

The means that have different superscript letters show a significant difference (p < 0.05).

The pH values of buffalo meat samples coated with different edible coatings increased over time (p < 0.05) (Table 8). The control group had the highest pH values at 6.04, followed by the LS group at 5.96, LS +0.5%PE at 5.86, LS +1.5%PE at 5.84, and LS +2.5%PE at 5.82, which had increasing amounts of PE. This pattern indicates that the edible coatings, especially those with higher PE concentrations, may have helped preserve the meat by preventing a rapid increase in pH. Microbial-based enzymatic activities have the potential to break down meat proteins into nitrogenous compounds with a basic nature such as trimethylamine and ammonia, which could subsequently increase the pH during storage [105]. However, edible coatings rich in PE could reduce microbial growth (Tables 2–4) and the synthesis of proteolytic enzymes, resulting in lower production of basic compounds and consequently a smaller change in pH in the meat samples.

These findings are consistent with a study by El-Saadony et al. (2021) [6], who used bioactive peptides in the form of coatings to extend the shelf life of raw buffalo meat.

The results presented in Table 8 demonstrate the variations in hardness values of buffalo meat coated with different edible coatings over different periods of storage. The hardness values showed a decrease from 58.88 N to 54.87 N as the storage time increased for all samples (p < 0.05). The control sample exhibited significantly lower hardness values of 54.65 N compared to the LS (56.43 N), LS + 0.5% PE (57.44 N), LS + 1.5% PE (58.13 N), and LS + 2.5% PE (58.35 N) samples at all storage times. Consequently, the LS + 2.5% PE coating was identified as the most effective in preserving the hardness of buffalo meat during storage. This effectiveness may be attributed to the phenolic extracts’ capability to delay the degradation of collagen and myofibrillar proteins by reducing the activity of microorganisms and endogenous enzymes (e.g., cathepsins, collagenases, and calpains) in the meat [18,106]. It was observed that a lower bacterial population corresponded to higher hardness values. The high hardness value observed in the LS + 2.5% PE (58.35 N) buffalo meat is likely due to a combination of factors, including reduced microbial activity, increased water holding capacity, and protein interaction. The antimicrobial properties of the PE may help preserve the meat’s texture by inhibiting spoilage, while enhanced water retention contributes to a firmer structure. Both mechanisms work together to create a product that is perceived as harder and more desirable in terms of texture. The coating may facilitate interactions between proteins and water, leading to a gel-like matrix that holds moisture within the meat. This structural change can contribute to a firmer texture, as the proteins are more effectively bound together [5,43,97,106].

Meat color is one of the most important visual factors and a key indicator for food consumption. Table 9 shows the color change of buffalo meat during different storage periods. The results of statistical analysis showed that there was no significant difference between the internal L* of meat samples coated with different PE (p>0.05). The control sample, when compared to the coated samples, showed a decrease in L* over time. While the coating samples containing internal PE exhibited a lower decreasing trend, attributed to the retention of PE during meat cultivation. In general, the L* values showed a decreasing trend during the storage period for all samples (p<0.05). Light scattering leads to the reduction of light reflection from the sample surface [1,107]. Mashau et al. (2022) [108] attributed the decrease in L* value to the presence of meat pigments, especially myoglobin. Additionally, Tanavar et al. (2021) [107] reported a decreasing trend in other L* color in the coated samples. During the 9-day storage period, a* decreased significantly (p>0.05) in all treatments. The same decreasing trend for bank a* was shown in the research of Karakosta et al. (2022) [1]. Naushad et al. (2022) [109] reported buffalo meat in the range of 5–12, consistent with a* (11.18–6.80) for buffalo meat in the current study. Many studies have also reported the reduction of meat’s red color due to the oxidation of oxymyoglobin (Fe^2+^) to methemoglobin (Fe^3+^) at low pressure [105,107,108]. According to the results of statistical analysis, banking b* decreased significantly in all samples on days 1 and 3 with different compositions; however, no significant difference was observed between days 6 and 9 (p > 0.05). Samples at the highest level showed that the coating and essential oil were higher in these samples, in agreement with the results of Noshad et al. (2022) [5] for buffalo meat and Tanavar et al. (2021) [107] for veal.

**Table 9:** Color change of buffalo meat as a function of storage time and edible coating type.

Parameters	Time (days)	Samples				
		Control	LS	LS+0.5%PE	LS+1.5%PE	LS+2.5%PE
L*	1	40.90 ± 0.51 a	37.28 ± 0.34 bcdef	38.30 ± 0.38 bcd	38.90 ± 0.43 abc	39.10 ± 0.33 ab
	3	36.60 ± 0.41 cdef	36.20 ± 0.40 defg	37.20 ± 0.40 bcdef	37.60 ± 0.46 bcde	37.80 ± 0.47 bcde
	6	32.50 ± 0.38 ij	33.47 ± 0.28 hi	35.24 ± 0.50 fgh	36.10 ± 0.43 defg	36.60 ± 0.50 cdef
	9	30.20 ± 0.29 j	32.29 ± 0.37 ij	33.60 ± 0.37 hi	34.10 ± 0.38 ghi	35.80 ± 0.46 efgh
					
a*	1	10.80 ± 0.32 ab	10.70 ± 0.33 ab	11.50 ± 0.50 a	11.40 ± 0.49 a	11.50 ± 0.28 a
	3	7.10 ± 0.37 cdef	7.80 ± 0.41 cde	8.80 ± 0.40 bc	8.90 ± 0.47 bc	9.10 ± 0.37 bc
	6	6.20 ± 0.46 def	7.10 ± 0.37 cdef	7.60 ± 0.37 cde	7.80 ± 0.36 cde	8.20 ± 0.42 cd
	9	5.10 ± 0.50 f	5.60 ± 0.26 ef	6.90 ± 0.39 cdef	7.10 ± 0.29 cdef	7.80 ± 0.50 cde
					
b*	1	6.50 ± 0.40 bcdef	7.50 ± 0.32 abcd	8.50 ± 0.60 ab	8.60 ± 0.43 ab	8.90 ± 0.42 a
	3	5.00 ± 0.26 efghi	6.10 ± 0.30 cdefg	7.30 ± 0.47 abcde	7.60 ± 0.49 abc	7.80 ± 0.29 abc
	6	3.60 ± 0.50 hi	4.10 ± 0.52 ghi	5.20 ± 0.39 defghi	5.80 ± 0.52 cdefgh	6.10 ± 0.37 cdefg
	9	3.20 ± 0.42 i	3.80 ± 0.41 ghi	4.20 ± 0.36 fghi	4.90 ± 0.39 fghi	5.20 ± 0.34 defghi
**ΔE**	1	-	-	-	-	-
	3	5.87 ± 0.04 fg	5.59 ± 0.07 g	4.28 ± 0.09 i	3.97 ± 0.01 ij	3.77 ± 0.04 j
	6	10.01 ± 0.02 b	8.64 ± 0.21 c	6.63 ± 0.01 e	5.71 ± 0.05 fg	5.04 ± 0.04 h
	9	12.57 ± 0.14 a	10.42 ± 0.21 b	8.59 ± 0.14 c	7.91 ± 0.18 d	6.07 ± 0.05 f
					
*h°*	1	31.01 ± 1.14 efg	35.02 ± 0.45 bcdef	36.43 ± 1.05 abcd	37.06 ± 0.32 abcd	37.72 ± 0.90 abc
	3	35.16 ± 0.01 bcde	38.04 ± 0.13 abc	39.52 ± 0.58 ab	40.47 ± 0.47 a	40.61 ± 0.13 a
	6	30.00 ± 2.30 fg	29.88 ± 2.62 g	34.65 ± 1.43 bcdefg	36.56 ± 1.70 abcd	36.63 ± 0.37 abcd
	9	32.02 ± 1.22 defg	34.05 ± 2.32 cdefg	31.28 ± 1.06 efg	34.55 ± 1.48 bcdefg	33.69 ± 0.05 cdefg
					
C*	1	12.60 ± 0.68 abc	13.07 ± 0.64 ab	14.30 ± 1.08 a	14.28 ± 0.92 a	14.54 ± 0.68 a
	3	8.68 ± 0.64 efgh	9.90 ± 0.72 bcdefg	11.43 ± 0.86 abcde	11.70 ± 0.96 abcde	11.99 ± 0.66 abcd
	6	7.17 ± 0.92 fgh	8.20 ± 0.82 fgh	9.21 ± 0.74 defgh	9.72 ± 0.85 cdefg	10.22 ± 0.80 bcdef
	9	6.02 ± 0.91 h	6.73 ± 0.70 gh	8.08 ± 0.72 fgh	8.62 ± 0.65 efgh	9.37 ± 0.85 defg
					

LS–*Lepidium sativum* mucilage; PE–propolis extract.

The means that have different superscript letters show a significant difference (p < 0.05).

Instrumentally measured color changes (ΔE) are considered visible changes to the human eye when the total color difference value exceeds 2 [110]. The sample ΔE was significantly higher than the coated samples (p < 0.05). Also, a significant difference (p < 0.05) was observed between the ΔE of the coated samples. Mashau et al. (2022) [108] and Noshad et al. (2022) [5] reported similar results, indicating the significant effect of ΔE coating on the essential oil of buffalo meat. Chroma index (C*) or saturation index shows the degree of saturation or color intensity [111]. The value of C* increased significantly with the addition of PE compared to the sample. The increase in C* in meat coating may be attributed to the increase in a* with the addition of essential oil. Results showed that C* decreased significantly during the storage period (p < 0.05), indicating that the samples’ color became lighter and more opaque over time. Therefore, the addition of internal essential oil improved the C* of the meat. Mashau et al. (2022) [108] reported similar results by adding moringa leaves to ground beef. Additionally, Sen et al. (2014) [111] obtained similar findings. Hue angle (*h°*) is a key point that shows the base color or dominant color in a sample. The closer *h°* is to 0, the purer and darker the red color sample becomes [111]. A significant increase in *h°* was observed in the coated samples (p < 0.05). The lower value of a* and C* and the higher value of *h°* indicate a change in meat color, associated with methemoglobin in the meat [108]. The incorporation of PE into the LS coating appeared to effectively inhibit significant color changes. Moreover, a higher concentration of PE in the coating corresponded to lesser color changes, signifying improved preservation of the meat’s color. This aligns with previous findings, which suggested that meat samples with minimal color changes due to coating may experience an extended shelf life [112].

#### 3.3.3. Sensory properties

As the storage time progressed, the sensory attributes of buffalo meat samples, including odor, color, texture, and overall acceptance, deteriorated (Table 10).

**Table 10:** Sensory properties of buffalo meat as a function of storage time and edible coating type.

Parameters	Time (days)	Samples				
		Control	LS	LS+0.5%PE	LS+1.5%PE	LS+2.5%PE
Color	1	8.80 ± 0.67 a	8.70 ± 0.74 a	8.40 ± 0.49 ab	8.50 ± 0.42 ab	8.50 ± 0.48 ab
	3	7.90 ± 0.58 abc	8.00 ± 0.66 ab	8.10 ± 0.57 ab	8.30 ± 0.48 ab	8.00 ± 0.42 ab
	6	5.50 ± 0.46 bcd	6.00 ± 0.55 abcd	6.20 ± 0.61 abcd	7.20 ± 0.53 abc	7.70 ± 0.53 abc
	9	3.40 ± 0.39 d	3.80 ± 0.47 d	4.90 ± 0.33 cd	6.30 ± 0.60 abcd	7.00 ± 0.50 abc
					
Odor	1	6.60 ± 0.44 ab	6.50 ± 0.29 abc	6.50 ± 0.40 abc	6.60 ± 0.57 ab	6.70 ± 0.37 a
	3	4.90 ± 0.49 abcde	5.10 ± 0.48 abcd	5.00 ± 0.45 abcd	5.50 ± 0.50 abcd	6.00 ± 0.40 abc
	6	3.80 ± 0.56 cdef	3.90 ± 0.63 bcdef	4.50 ± 0.55 abcdef	4.90 ± 0.42 abcde	5.10 ± 0.52 abcd
	9	1.80 ± 0.59 f	2.00 ± 0.47 f	2.20 ± 0.57 ef	2.90 ± 0.36 def	3.10 ± 0.48 def
					
Texture	1	8.80 ± 0.29 a	8.70 ± 0.26 a	8.50 ± 0.42 a	9.00 ± 0.50 a	9.00 ± 0.37 a
	3	7.50 ± 0.34 abcd	7.20 ± 0.38 abcd	7.60 ± 0.33 abcd	8.20 ± 0.48 ab	8.60 ± 0.30 a
	6	7.00 ± 0.40 abcd	7.00 ± 0.50 abcd	7.30 ± 0.41 abcd	7.90 ± 0.39 abc	8.40 ± 0.50 a
	9	5.20 ± 0.49 d	5.50 ± 0.66 cd	5.90 ± 0.46 bcd	7.50 ± 0.40 abcd	7.90 ± 0.53 abc
					
Overall acceptance	1	8.50 ± 0.47 a	7.90 ± 0.36 abc	8.20 ± 0.68 ab	8.30 ± 0.50 ab	8.40 ± 0.44 a
	3	5.20 ± 0.40 cde	5.60 ± 0.39 bcde	6.70 ± 0.52 abcd	7.00 ± 0.43 abcd	7.10 ± 0.30 abcd
	6	3.50 ± 0.53 e	3.90 ± 0.58 e	4.50 ± 0.47 de	4.80 ± 0.57 de	5.00 ± 0.50 de
	9	3.10 ± 0.51 e	3.30 ± 0.50 e	3.60 ± 0.40 e	3.80 ± 0.38 e	3.90 ± 0.37 e

LS–*Lepidium sativum* mucilage; PE–propolis extract.

The means that have different superscript letters show a significant difference (p < 0.05).

However, the coated samples, particularly those with PE, exhibited significantly higher sensory scores than the control sample. It’s important to note that meat samples are generally considered acceptable for consumption when they receive high scores (> 4) [18]. In terms of overall acceptance, the control and LS samples were deemed unacceptable after 3 days of storage; however, the LS+0.5%PE, LS+1.5%PE, and LS+2.5%PE samples were acceptable at 6 days of storage. This implies that the addition of PE to the edible coating extended the shelf life of buffalo meat by three days. Consistent with our findings, Shavisi et al. (2017) [113] reported that films containing 2% *Ziziphora clinopodioides* essential oil alone and in combination with different concentrations of PE and cellulose nanoparticle extended the shelf life of minced beef for at least 11 days under refrigerated conditions without any unfavorable organoleptic properties. Additionally, the incorporation of ethanolic extract of propolis into xanthan coating has been found to be more effective in significantly preserving (p < 0.05) the taste and odor of coated fish fillet samples compared to the control [114].

### 3.4. Results of modelling

Table 11 shows an analysis of the performance metrics for the SVM and RBF methodologies, assessed during the training, testing, and total stages through the application of three recognized statistical indicators.

**Table 11:** Statistical analysis of SVM and RBF (one hidden layer with Trainbr algorithem) models for predicting all the parameters.

Model	Parameters	Train		Test		Total	
		MAPE	R^2^	MAPE	R^2^	MAPE	R^2^
**SVM**	pH	2.12	0.97	2.67	0.96	2.23	0.98
	Overall acceptance	1.36	0.98	1.43	0.98	1.30	0.98
	Moisture	1.97	0.97	2.01	0.97	1.99	0.97
	Total viable count	1.86	0.98	1.89	0.97	1.83	0.98
	ΔE	2.94	0.96	2.53	0.97	2.24	0.96
	Fungi	3.52	0.96	3.73	0.96	3.53	0.96
	Psychrotrophic bacteria count	1.78	0.98	1.82	0.98	1.80	0.97
	Coliforms	2.67	0.98	2.74	0.97	2.56	0.97
	Hardness	2.90	0.98	2.86	0.98	2.80	0.98
						
	**Parameters**	**Train**		**Test**		**Test**	
		MAPE	R^2^	MAPE	R^2^	MAPE	R^2^
**RBF**	pH	1.23	0.98	1.32	0.98	1.20	0.98
	Overall acceptance	1.79	0.97	1.82	0.97	1.75	0.97
	Moisture	0.62	0.99	0.76	0.99	0.65	0.99
	Total viable count	1.13	0.98	1.21	0.98	1.15	0.98
	Fungi	1.95	0.97	2.02	0.96	1.97	0.97
	Psychrotrophic bacteria count	1.56	0.98	1.64	0.98	1.60	0.98
	Coliforms	1.86	0.98	1.90	0.98	1.87	0.98
	ΔE	2.31	0.95	2.76	0.95	2.19	0.95
	Hardness	1.76	0.98	1.83	0.97	1.80	0.98

The findings indicate a superior performance by the RBF model over the SVM in predicting the accuracy of the output factors. Notably, the RBF model displayed MAPE values from as low as 0.65% for moisture content up to 2.19% for ΔE, whereas the SVM model showed a range from 1.30% for overall acceptance to 3.53% for fungi. As a result, the SVM model proves to be a reliable predictor for all 9 parameters under review, offering a possibility to lessen dependence on expensive laboratory tests. Additionally, the adaptability of the RBF model enables its application alongside diverse sensory technologies, such as electronic noses, tongues, computer vision systems, and near-infrared spectroscopy, to provide a comprehensive assessment of food qualities. Further research conducted by Zibaei-Rad et al 2023 [115], emphasized the effectiveness of ANN in forecasting the viability of probiotics, taking into account varying pH levels and time periods. In another similar study, SVM with different algorithms, including linear, polynomial, and RBF, along with other classification algorithms used for evaluating dry bean properties. The results showed that SVM with RBF kernel algorithm achieved the highest accuracy. This study emphasizes the importance of selecting appropriate SVM algorithms for complex and non-linear structured datasets, which is in line with the superior performance of the RBF model over the SVM in your findings. These results presenting an economical substitute to elaborate lab examinations.

## 4. Conclusions

The quality and shelf life of meat and meat products could be significantly affected by microbial growth. The PE included gallic acid (98.593), benzoic acid (119.972), syringic acid (91.654), 4–3 dimethoxy cinnamic acid (323.534), *p*-coumaric acid (492.698), myricetin (90.277), caffeic acid (159.184), luteolin (143.543), chlorogenic acid (105.333), and apigenin (100.431). The PE was determined to have a TPC of 36.67 ± 0.57 mg GAE/g and a TFC of 48.02 ± 0.65 mg QE/g. The extract’s radical scavenging activity ranged from 0 to 76.22% for DPPH radicals and from 0 to 50.31% for ABTS radicals. The PE contained numerous bioactive compounds that exhibited excellent and antimicrobial properties. When incorporated into the LS-based edible coating, the PE significantly reduced microbial growth in buffalo meat compared to the coating without the extract. The bioactive-infused edible coating also minimized weight and texture losses during display and enhanced the overall acceptability of the meat. In the conducted research, a comparative assessment was performed to ascertain the predictive capabilities of SVM and RBF models concerning nine unique variables obtained from laboratory tests. The results underscored the enhanced performance of the RBF model in comparison to the SVM. The RBF model demonstrated remarkable predictive accuracy, consistently delivering precise results even when applied to datasets of a smaller scale. The results indicated that the effect of LS+ 2.5% PE wrapping on buffalo meat was to retain their good quality characteristics and extend the shelf life during storage, which was supported by the results of microbiological, physical, and sensory evaluation. Thus, LS containing PE could be used as an active packaging to maintain quality and extend the shelf life of the buffalo meat at 4°C. Therefore, this type of food coating, renowned for its strong antimicrobial properties, has the potential to effectively package and preserve perishable and delicate food items, such as meat.

## Supporting information

S1 Data (XLSX)
